# Mechanisms of immune evasion in bladder cancer

**DOI:** 10.1007/s00262-019-02443-4

**Published:** 2019-12-06

**Authors:** Paul L. Crispen, Sergei Kusmartsev

**Affiliations:** Department of Urology, University of Florida, College of Medicine, 1200 Newell Dr, PO BOX 100247, Gainesville, FL 32610 USA

**Keywords:** Bladder cancer, Cancer immunotherapy, Immune tolerance, Immune evasion, Tumor microenvironment

## Abstract

With the introduction of multiple new agents, the role of immunotherapy is rapidly expanding across all malignancies. Bladder cancer is known to be immunogenic and is responsive to immunotherapy including intravesical BCG and immune checkpoint inhibitors. Multiple trials have addressed the role of checkpoint inhibitors in advanced bladder cancer, including atezolizumab, avelumab, durvalumab, nivolumab and pembrolizumab (all targeting the PD1/PD-L1 pathway). While these trials have demonstrated promising results and improvements over existing therapies, less than half of patients with advanced disease demonstrate clinical benefit from checkpoint inhibitor therapy. Recent breakthroughs in cancer biology and immunology have led to an improved understanding of the influence of the tumor microenvironment on the host’s immune system. It appears that tumors promote the formation of highly immunosuppressive microenvironments preventing generation of effective anti-tumor immune response through multiple mechanisms. Therefore, reconditioning of the tumor microenvironment and restoration of the competent immune response is essential for achieving optimal efficacy of cancer immunotherapy. In this review, we aim to discuss the major mechanisms of immune evasion in bladder cancer and highlight novel pathways and molecular targets that may help to attenuate tumor-induced immune tolerance, overcome resistance to immunotherapy and improve clinical outcomes.

## Introduction

Bladder cancer is the ninth most common malignancy worldwide and the fifth most common in developed countries. Approximately 20% of patients are diagnosed with muscle-invasive disease at the time of initial presentation, which will require multiple treatment modalities due to the high rates of disease recurrence, progression and disease-specific mortality. Treatment options include chemotherapy, radiation therapy, and radical cystectomy in cases of clinically localized disease and systemic chemotherapy for patients with metastatic disease. Despite this aggressive treatment approach prognosis remains poor for many patients. The continued poor prognosis observed presents an opportunity for immunotherapy to improve outcomes. During the past two decades, several revolutionary immunotherapy approaches have taken center stage in cancer therapy. These approaches include checkpoint inhibitors PD-L1/PD1, CTLA-4 as well as CAR T cell therapy [1–3]. Anti-PD-L1/PD1 and anti-CTLA-4 therapies that are based on antibody treatment have shown significant clinical effects in various solid cancers, including bladder cancer. However, there is still an unmet need, as the majority of patients do not respond to the immunotherapy in all stages of bladder cancer. A greater understanding of the mechanisms of resistance to immunotherapy may provide alternate strategies to improve bladder cancer care. In this review, we discuss the current use and limitations of immunotherapy in bladder cancer and explore various mechanisms of resistance to immunotherapy, which may serve as future therapeutic targets.

## Immunotherapy for bladder cancer

### Bacillus-Calmette–Guerin

Intravesical Bacillus-Calmette–Guerin (BCG) was first approved for use in the United States in 1990 for stage I bladder cancer. Currently, it is the most common form of immunotherapy used for bladder cancer. BCG induces an initial complete response rates of 55–70% in patients with high-risk stage I bladder cancer. Conversely, despite high initial success rates, as many as 25–45% of patients will not respond, and an additional 40% of patients will eventually relapse despite showing initial success [4]. While the exact mechanism of action remains unknown, BCG is known to induce a robust innate immune response leading to long-lasting adaptive immunity [5]. The inciting events leading to this immune response may involve multiple pathways including BCG attachment to and internalization within the urothelium. The process of BCG attachment to the urothelium has been widely studied with inconsistent results on its importance to the efficacy of treatment. Similarly, BCG internalization into the urothelium may be possible but is likely only transient with decreasing mycobacterial DNA being detected in the urine overtime following instillation. Regardless of the manner of induction, BCG stimulates an innate immune response locally and systemically. Following initial instillation cytokine and chemokine concentrations peak within 2–8 h leading to immune cell recruitment to the urothelium. The roles of neutrophils, natural killer (NK) cells, CD8^+^ T cells, and macrophages have all been explored individually with all of these cells appearing to be important in the initial response. This innate response is further characterized by granuloma formation in the bladder wall, containing macrophages, dendritic cells (DCs), lymphocytes, neutrophils and fibroblasts [6, 7]. Induction of adaptive immunity also appears critical for the success of BCG therapy. The importance of T cells in the response to BCG has been clearly demonstrated in both animal and human studies [5–7]. Furthermore, the importance of adaptive immunity is supported with improved 5-year disease-free survival of 80% patients with a positive PPD test prior to the initiation of BCG therapy compared to only 45% in patients who were PPD negative prior to the initiation of BCG therapy [8]. Enhancing the immune response to BCG may further improve patient outcomes. While the initial trial evaluating BCG vaccination with intravesical therapy did not show clinical benefit, ongoing clinical trials may provide greater insight into the importance of the adaptive immune response due to the timing and manner of BCG vaccination. [9, 10].

### Immune checkpoint blockade

Immune checkpoint blockade (ICB), including anti-PD1/PD-L1 and anti-CTLA-4 therapies has shown tremendous success in the treatment of human cancers, particularly for solid tumors. Cancers with high mutational burden including Hodgkin’s lymphoma, melanoma, renal cell carcinoma, non-small lung cancer carcinoma, urothelial bladder carcinoma have all demonstrated promising response rates to anti-PD1/PD-L1 antibody therapies [11–16].

Multiple studies have demonstrated that blocking PD-1 or its ligand, PD-L1, result in encouraging rates of anti-tumor activity in patients with metastatic urothelial cancer who had disease progression following standard chemotherapy. Currently, the United States Food and Drug Administration has approved two PD1 inhibitors (Nivolumab and Pembrolizumab) and three PD-L1 inhibitors (Atezolizumab, Avelumab, and Durvalumab) for the treatment of advanced urothelial carcinoma. The overall response rates noted in clinical trials leading to approval ranged from 15 to 29% across the approved agents. Importantly, trials that randomized patients to PD1/PD-L1 inhibition versus investigator choice of single-agent chemotherapy demonstrated remarkable improvements in side effect profile and survival. IMvigor 211, sponsored by Hoffman-La Roche, was a randomized phase III trial comparing Atezolizumab versus investigator choice single-agent chemotherapy in patients with disease progression/recurrence after platinum-based chemotherapy [17]. While no advantage was noted with Atezolizumab in overall response rate, 23% versus 21%, and overall survival, 11.1 months versus 10.6 months, the side effect profile was significantly more favorable in patients receiving Atezolizumab [18]. Treatment-related adverse effects, 69.5% versus 89.2%, and treatment-related grade 3–4 adverse effects were significantly lower in patients receiving Atezolizumab compared to single-agent chemotherapy. Keynote-045, sponsored by Merck, randomized patients with disease progression/recurrence following platinum-based chemotherapy to Pembrolizumab versus investigator choice single agent choice chemotherapy. Patients receiving Pembrolizumab demonstrated a significant increase in overall response rate, 21% versus 11%, and median overall survival, 10.3 months versus 7.4 months. Additionally, the number of adverse events and the severity of adverse events were lower in patients receiving Pembrolizumab. While these trials suggest that PD1/PD-L1 inhibitors have favorable side effect profiles compared to chemotherapy, side effect profiles vary between individual PD1/PD-L1 inhibitors. Factors believed to influence the variation in side effect profile between individual PD1/PD-L1 inhibitors include the primary site of cancer being treated and selectivity of the antibodies [19]. With the favorable results from multiple trials, the National Comprehensive Cancer Network guidelines now recommend Pembrolizumab as a preferred regimen in patients with disease progression/recurrence of locally advanced or metastatic urothelial carcinoma following platinum-based chemotherapy. Alternative preferred regimens for this patient population include Atezolizumab, Nivolumab, Durvalumab, and Avelumab [20, 21]. For patients that are not eligible for cisplatin as first-line therapy, Atezolizumab and Pembrolizumab are preferred first-line regimens.

Although the initial results of trials evaluating PD1/PD-L1 inhibition in advanced urothelial carcinoma are promising, a large majority of patients do not respond to anti-PD-L1 antibody monotherapy. Investigations exploring the potential value of biomarkers predicting treatment response are ongoing. To date, multiple biomarkers have been evaluated to predict response to PD1/PD-L1 inhibition in urothelial carcinoma. PD1 expression within the tumor has been associated with an increased overall response rate in multiple trials, however, patients with tumors without PD1 expression have also demonstrated response limiting this biomarker for treatment selection. Higher tumor mutation burden, interferon-gamma gene expression, and DNA damage repair alterations have also been associated with response rates but require additional validation.

However, not all tumors express PD-L1 and display immune infiltration (“cold tumor microenvironment”). In this case, combination of conventional therapy, including chemotherapy, radiation and immune checkpoint inhibitors may improve the response rates, as tumor cell death initiated by radiation or chemotherapy may release tumor-antigens, attract antigen-presenting cells and stimulate a T cell-mediated anti-tumor immune response. The resulting immune response may be further enhanced by applying checkpoint inhibitors [22].

## Mechanisms of immune evasion in bladder cancer

Bladder cancer represents an ideal disease state to study immune evasion and mechanisms by which to improve the immune response based on several established features. These features include distinct molecular/genomic subtypes of bladder cancer, known response rates to currently available immune therapy, and unique opportunity to study treatment response. Altered signaling pathways and protein expression in bladder cancer include, but are not limited to the p53/cell cycle, DNA repair, PI3K/AKT, and chromatin modifications. Molecular subtypes can be categorized based on these alterations which show differing response rates to chemotherapy clinically, but also demonstrate associations within the tumor microenvironment including the degree of inflammation and distribution of tumor-infiltrating lymphocytes [23]. The amount of inflammation and tumor-infiltrating lymphocytes has been associated with overall survival, presenting a potential opportunity for initiating the immune response in tumors with low levels of inflammation. Additionally, The Cancer Genome Atlas (TCGA) study found that genes regulating chromatin remodeling are more frequently mutated in bladder cancer than in other type of cancer, which may represent an additional target for novel therapies to be given in combination with immunotherapy [24]. Furthermore, bladder cancer is associated with one of the highest mutation burdens among all types of cancer which is a known predictor of treatment response to checkpoint inhibitors [24]. Additionally, neoantigens produced from cancer somatic mutations are positively associated with response to anti-PD-1 or anti-CTLA-4 treatment [25]. These mutations may also add more complexity to the tumor microenvironment, regulating expression of inhibitory or stimulatory molecules. Lastly, bladder cancer provides a unique opportunity to study treatment response due to the ability to access the bladder for repeat resections of primary tumors and administration of intravesical therapy. These features can allow for a minimally invasive manner to assess treatment response and evaluate new therapies while potentially decreasing systemic toxicity by limiting exposure of the treatment to the bladder surface.

### Immunosuppressive tumor microenvironment

Despite the success of immunotherapy in the treatment of bladder cancer, there remains a tremendous opportunity to improve response rates and prediction of treatment response. Mechanisms of immune evasion in bladder cancer beyond PD1/PD-L1 expression may offer additional therapeutic targets to improve patient outcomes. Tumors evade immune surveillance through multiple mechanisms. The ability to escape the immune system is an important characteristic of malignant cells, which enhances tumor survival, proliferation and dissemination. Therefore, identifying specific mechanisms of immune evasion could improve the efficacy of existing cancer immunotherapies by removal tumor-induced immunosuppression and boosting the host’s immune response against tumors.

**Fig. 1:**
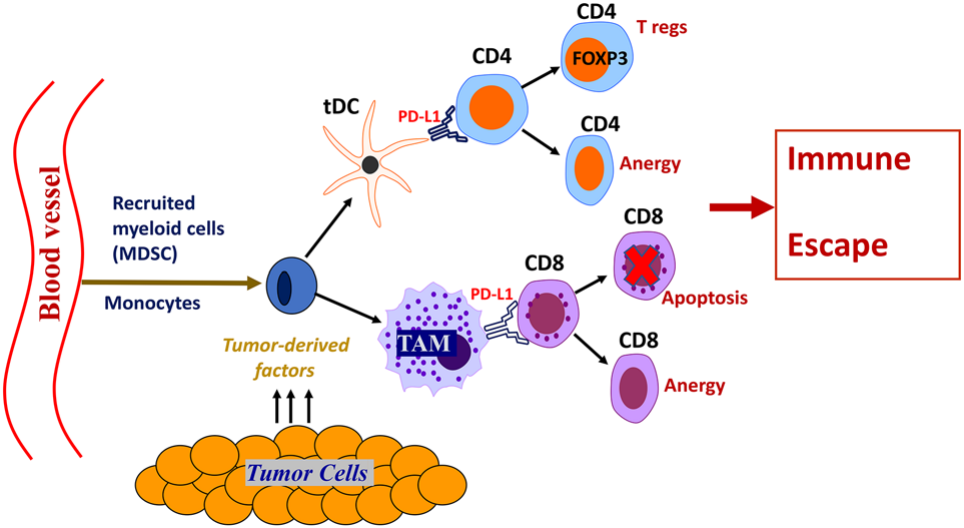
Tumors recruit immunosuppressive MDSCs and tolerogenic T regs. Malignant cells constantly secrete significant amounts of chemoattractants, such as CCL2, CCL18, and CCL1 that support cancer-related inflammation and stimulate recruitment of MDSCs to the tumor’s vicinity. MDSCs give rise to the development of tDCs and PD-L1^+^ TAMs that migrate through lymphatics to draining lymph nodes and stimulate the generation of T regs. Accumulation of T regs and MDSCs promote immune suppression in the tumor microenvironment

**Fig. 2:**
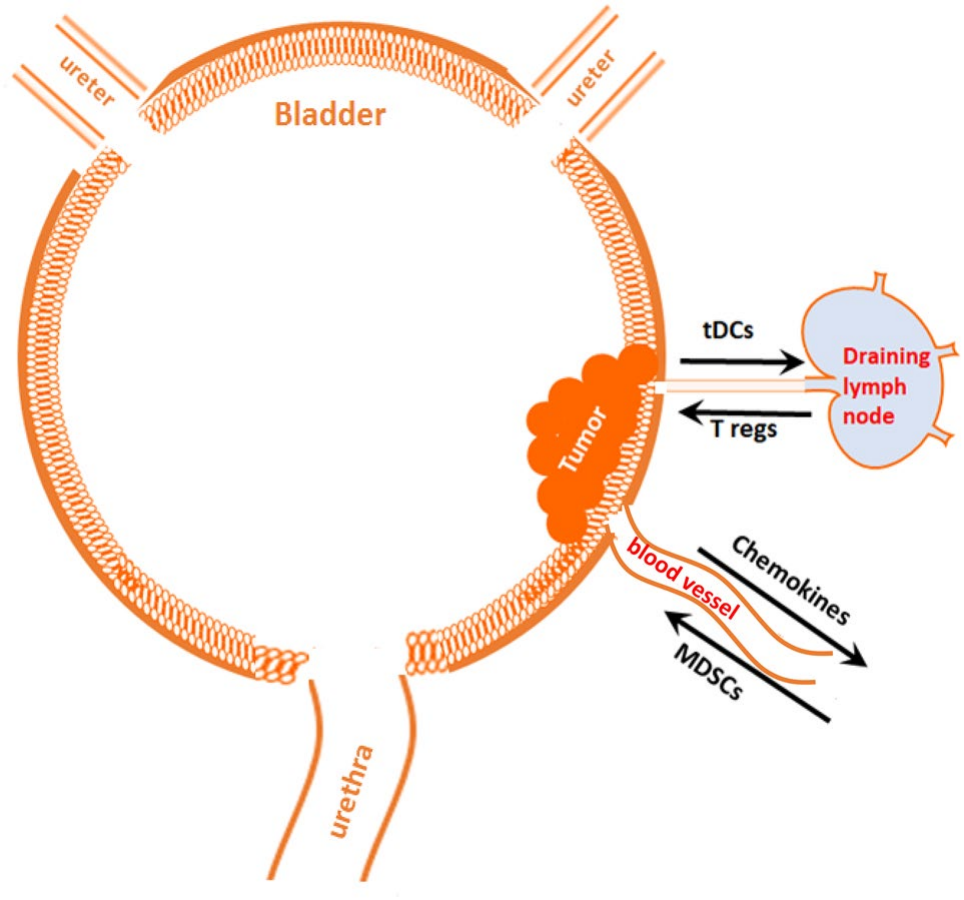
Bladder tumor-induced immune suppression promotes the escape of malignant epithelial bladder cells from the immune system. Upon entering tumor tissue, the myelomonocytic cells such as MDSCs, depending on local milieu, differentiate into PD-L1^+^ tDCs or PD-L1^+^ TAMs. These PD-L1-expressing APCs are immunosuppressive and capable of induction T cell anergy and/or T cell apoptosis in an antigen-specific manner. Inability of the host’s immune system to generate an effective T cell-mediated anti-tumor immune response results in tumor protection and promotes further tumor growth

One of the hallmarks of cancer progression is a formation of immunosuppressive and tolerogenic tumor microenvironment [26]. Tumor tissue itself is highly heterogeneous, dynamic and consists of epithelial tumor cells, immune infiltrating cells, vascular cells, stromal cells and an extracellular matrix (ECM). To develop an effective anti-tumor immune response that will result in immune-mediated tumor eradication, a concerted effort of antigen-presenting cells (APCs) (DCs, macrophages), lymphocytes (CD8, CD4) and NK cells is required. To survive and escape the normal response of these immune cells, tumors secrete various immunosuppressive and anti-apoptotic factors including TGF-beta, PGE2, IL-10, and IL-6 [26–29], creating a highly tolerogenic microenvironment. In addition, the tumor microenvironment is tightly linked to the accumulation of several types of immune cells with immunosuppressive phenotypes such as myeloid-derived suppressor cells (MDSCs), tolerogenic DCs (tDCs), tumor-associated macrophages (TAMs) and regulatory T cells (T regs). These changes have all been noted in bladder cancer (Figs. 1, 2), which is characterized by a highly immunosuppressive microenvironment that includes increased expression of the inhibitory ligand PD-L1, the strong presence of MDSCs, TAMs, increased PGE2 production and aberrant metabolism of glycosaminoglycans such hyaluronic acid or hyaluronan.

### PD-L1/PD1 pathway

The immunosuppressive ligand PD-L1 can be expressed by tumor cells and by the host’s myeloid cells. Since PD-L1-expressing cells can induce apoptosis or anergy of activated T lymphocytes through binding of PD-L1 to cognate receptor PD1 (CD279) on T cells, PD-L1-mediated inhibition of activated PD1^+^ T lymphocytes is considered a major mechanism for tumor immune escape [30]. Blockade of PD-L1/PD1 signaling attenuates tumor-induced immune suppression and successfully inhibits tumor growth due to preservation of a T cell mediated anti-tumor immune response. Recent studies demonstrated that PD-L1 expression on host APCs, such as macrophages and DCs, is essential for PD-L1 mediated immune evasion [31, 32]. We recently reported that bone marrow-derived myeloid progenitors, including MDSCs, differentiate into highly immunosuppressive PD-L1^+^ macrophages upon contact with tumor cells [33].

Interestingly, many human or murine tumor cell lines do not express PD-L1constitutively, but at the same time, most of surgically removed tumors demonstrate high expression of PD-L1 [30, 33]. This finding may suggest that inflammatory or immune cells recruited to the malignant tissues, could be involved in the mechanisms of PD-L1 expression induction within the tumors. Additionally, cytokines and other immunosuppressive factors secreted by tumor-recruited myeloid cells play multifaceted roles in mechanisms of regulation of PD-L1 expression.

### Myeloid-derived suppressor cells

Accumulation of MDSCs in the tumor microenvironment has been extensively reported in various experimental models and in human tumors. MDSCs are a heterogeneous population of immature myeloid cells that are recruited to the primary tumor as well as metastatic sites and play a crucial role in inhibiting innate and adaptive immune responses by suppressing CD4 T-cells, CD8 T-cells, and NK cells [34]. In the clinical setting, an increased number of MDSCs correlates with weakened clinical responses to immunotherapy. Moreover, low levels of circulating or tumor-infiltrating MDSCs have been attributed to an improved prognostic and predictive value in a variety of oncologic settings [35]. Multiple studies have shown that CD11b-expressing MDSCs play a profound role in suppressing the antigen-specific T cell response. Morphological analyses have shown that MDSCs are comprised of myeloid cells and their precursors at various stages of differentiation. The two major myeloid cell subsets of MDSCs, monocytic and granulocyte-type cells, are detected in peripheral blood of cancer patients [34, 36]. In the presence of specific growth factors and/or cytokines, monocytic MDSCs can differentiate into mature DCs or macrophages in vitro as well as in vivo. However, tumor-derived products prevent this developmental pathway favoring formation and accumulation of immunosuppressive MDSCs [34].

Patients with bladder cancer have an increased amount of MDSCs in peripheral blood compared to healthy donors [37, 38]. These MDSCs are represented by both cell subsets: granulocytic CD15^high^ CD33^low^ HLA-DR^neg^ and monocytic CD15^low^ CD33^high^ HLA-DR^neg^ MDSCs. Cytokine/chemokine profiling of these MDSCs demonstrated that both myeloid cell subsets from cancer patients produced substantial amounts of CCL2, CCL3, CCL4, G-CSF, IL-8 and IL-6. Furthermore, isolated granulocytic MDSCs exhibited immunosuppressive activity by inducing CD4^+^Foxp3^+^ T regs cells and inhibiting the T cell proliferative response. Furthermore, analysis of tumor tissue obtained from patients with bladder cancer showed significant presence of intra-tumoral myeloid cells including HLA-DR-negative MDSCs and HLA-DR-positive TAMs. Similarly, bladder tumor-infiltrating MDSCs consisted of the two major cell subsets: monocytic and granulocytic [37]. Altogether, these data demonstrate that human bladder cancer is associated with an increased number of myeloid cells in both the peripheral blood and tumor tissue. Those myeloid cells secrete significant amounts of pro-inflammatory and immunosuppressive cytokines/chemokines which contributes to cancer-related inflammation and immune evasion.

Additional data on the importance of the interaction between the bladder cancer microenvironment and myeloid cells has been demonstrated in vivo. Human bladder cancer cells implanted into immunodeficient mice become quickly infiltrated with the host’s myeloid cells, including MDSCs and macrophages [39]. Fast-growing SW780 tumors are characterized by more active recruitment of myeloid cells into tumor tissues (up to 40% of total tumor cell population). Those SW780 tumor-infiltrating myeloid cells were mostly represented by MHC class II-positive F4/80^+^ macrophages and Ly6C^+^F4/80^+^ macrophage precursors. Slow-growing Urothel 11 bladder tumors were less capable in the recruitment of myeloid cells into the tumor bed (up to 25% of total tumor cell population). Analysis of intra-tumoral myeloid cells showed that the majority of these cells displayed an immature phenotype including Ly6c^+^F4/80^−^ MDSCs and Ly6C^+^F4/80^+^ macrophage precursors. This data suggests that tumor growth rate and tumor size may influence the tumor’s ability: (1) to recruit myeloid cells and (2) convert recruited myeloid cells into tumor-promoting TAMs.

Using an experimental mouse model of bladder cancer, we demonstrated that Gr-1^+^ MDSCs isolated from the spleen of MBT2-tumor bearing mice or naïve bone marrow are able to differentiate into highly immunosuppressive PD-L1^+^ macrophages upon contact with bladder cancer cells [32]. This tumor-mediated PD-L1 expression in myeloid cells was dependent on PGE_2_ production, since in vitro and in vivo inhibition of PGE_2_ synthesis with pharmacological inhibitors markedly reduced PD-L1 by myeloid cells. PD-L1^+^ cells isolated from tumor tissue also displayed the morphology of TAMs and expressed high levels of PGE_2_-forming enzymes mPGES1 and COX2.

### Tumor-associated macrophages

**Fig. 3:**
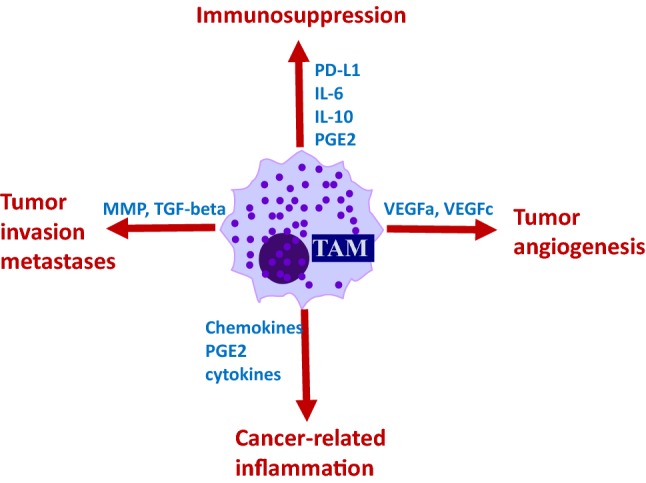
TAMs play multifaceted roles in tumor development and progression. TAMs arise from tumor-recruited blood monocytes or myeloid progenitors including MDSCs. Tumor microenvironment and local cytokine milieu promote polarization of TAMs making them immunosuppressive and tumor-supporting cells. TAMs are abundant in tumor stoma. These cells secrete multiple factors that promote tumor growth via stimulation of tumor angiogenesis, tumor invasion, inflammation and immune escape

TAMs are considered one of the major players in the regulation of the immune responses in cancer (Fig. 3) and are abundant in the tumor stroma at all stages of cancer progression. Macrophages are known to contribute to metastasis by priming the pre-metastatic site and enabling tumor cell extravasation and survival [40, 41]. Furthermore, it has been shown that TAMs orchestrate angiogenesis by secreting pro-angiogenic molecules that increase the tumor vascular density and promote a chronic inflammatory environment permissive for tumor initiation and growth through the release of inflammatory cytokines. Additionally, extensive TAM infiltration positively correlates with cancer metastasis and poor clinical prognosis in a variety of human cancers [40, 41].

TAMs can differentiate from circulating blood CD14 monocytes or from tumor-recruited MDSCs. Phenotype, cytokine profile and immune function of these cells are tightly regulated by the tumor microenvironment. Both clinical studies and experimental mouse models indicate that TAMs are typically polarized by the local tumor milieu to adopt pro-tumoral characteristics and frequently acquire a M_2_ phenotype in tumor microenvironment. TAMs greatly contribute to the formation of a tolerogenic tumor microenvironment by direct elimination of CD8 T cells [42, 43], supporting induction and trafficking of T regs [44–46] and secreting immunosuppressive cytokines and bioactive lipids [47, 48]. TAMs frequently express PD-L1 and are capable of inhibiting the function of tumor-infiltrating CD4 and CD8 T cells through PD-L1/PD1 interaction [11, 33, 49]. In bladder cancer, increased infiltration of TAMs is associated with poor prognosis after intravesical instillation of BCG [50]. Moreover, the predominance of M2-polarized TAMs in the stroma of low-hypoxic bladder tumors is frequently associated with resistance to BCG immunotherapy. Some studies suggest that TAMs may directly interfere with the BCG-induced immune response and may represent a surrogate marker for BCG resistance [51].

### Tolerogenic dendritic cells

DCs are a vital component of the immune system and are required for the generation of an anti-tumor immune response. These cells are designed to pick up tumor antigens and present them to T lymphocytes in an immune-stimulating manner thus triggering development of an adaptive immune response [52]. However, tumors can have a significant influence on the differentiation/maturation process of DCs, preventing the up-regulation of co-stimulatory molecules required for the immune-stimulating function and converting them into tDCs. The balance between the levels of expression of co-stimulatory relative to co-regulatory molecules at the APC surface is a crucial determinant of the outcome of DC-T cell interaction; higher relative expression of co-stimulatory molecules is predictive of T-cell activation, while higher expression of some immunosuppressive molecules, particularly PD-L1, correlates with suppression of the immune response [53]. tDCs have been observed in bladder tumors [54]. The tumor-secreted factors involved in this process include IL-10, PGE_2_, VEGF, TGF-β and other tolerogenic factors [53, 55]. Phenotypically, these cells retain expression of the monocyte/macrophage marker CD14, show lack of CD1a, but up-regulate the immunosuppressive ligand PD-L1. PD-L1 expression in tDCs is controlled by the transcriptional factor STAT3 [56]. Functionally, interaction of tolerogenic PD-L1^+^ DCs with PD1^+^ CD4 or CD8 T cells results in T cell anergy and/or induction of T regs.

### Regulatory T cells

Previously published studies have identified a subset of T cells, which act in a regulatory capacity by suppressing the function of other T cells [57, 58]. The role of T regs is to dampen chronic immune responses against viruses, tumors and self-antigens. T regs develop from circulating CD4 T helper cells in response to antigen-specific presentation by tDCs or by TAMs and are characterized by expression of the transcription factor FOXP3. A common trait of all T regs is the expression of one or more anti-inflammatory molecules, such as IL-10, TGFβ or IL-35 and/or inhibitory receptors such as CTLA-4, LAG-3, GITR among others [59]. Accordingly, elevated levels of T regs in the peripheral blood and accumulation within tumor tissue and lymph nodes have been detected in patients with urothelial carcinomas and other malignancies [60, 61]. In a recently published study, the level of T regs in human bladder tissue significantly correlated with both TAMs and with IL-6-positive cancer cell count [50]. FOXP3^+^ CD4^+^ T regs accounted for over 20% of the CD4^+^ T-cell population. Bladder cancer-infiltrating CD4^+^FOXP3^+^ T cells did not produce IL-2 or IFN-γ even upon stimulation, and readily suppressed autologous CD4^+^ effector T cells, confirming that tumor-infiltrating CD4^+^FOXP3^+^ T cells act functionally as T regs. Similar functional findings have been reported for other solid tumors, including melanoma and ovarian cancer.

### PGE_2_ metabolism and immune evasion

Due to the high expression of the inducible inflammatory enzyme COX2, bladder carcinoma tissues secrete substantial amounts of PGE_2_ [39, 62]. Endogenously released PGE_2_, the major metabolite of the COX pathway, plays multifaceted roles in cancer progression, cancer-related immune inflammation and immune evasion. This lipid metabolite exhibits strong anti-apoptotic effects, supports proliferation and renewal of bladder cancer stem cells and induces resistance to chemotherapy [63]. In regard to regulation of the immune response, it is reported that PGE_2_ inhibits antigen-presenting cell differentiation [64–67], stimulates expression of arginase I in myeloid cells and promotes accumulation of MDSCs in a dose-dependent manner [68, 69]. Furthermore, PGE_2_ promotes recruitment of T regs to the tumor site and induces Foxp3 in T cells [70, 71]. This lipid metabolite also inhibits the NK-mediated cytotoxic activity against tumors and transactivates the M-CSF1 receptor and synergizes with colony-stimulating factor-1 in the induction of M2 macrophages via the mitogen-activated protein kinase ERK1/2 [72]. PGE_2_ strongly suppresses IFN-γ and IL-2 production by T cells and NK cells while enhancing IL-4 and IL-10 production, thereby promoting a Th_2_ over Th_1_ immune response [73, 74]. Furthermore, inhibition of PGE_2_ secretion during the induction of Ag-specific immunity results in enhanced immune and therapeutic activity of cancer vaccines [75].

In addition to tumor cells, myeloid cells that infiltrate tumor tissue also demonstrate increased PGE_2_ production via upregulated expression of COX2 and mPGES1 [33, 76]. Elevated levels of PGE_2_ in the tumor microenvironment in combination with other tumor-derived factors affect the immune function of APCs by driving their differentiation toward immunosuppressive PD-L1-expressing macrophages [33]. Increased expression of COX2/mPGES1 in myeloid cells was associated with driving the differentiation of macrophages toward the M2 phenotype characterized by increased arginase activity and production of pro-tumoral factors such as IL-10, VEGF and CXCL2. Notably, tumor-infiltrating myeloid cells were also characterized by down-regulated expression of the major PGE_2_-catabolizing enzyme 15-hydroxyprostaglandin dehydrogenase (15-PGDH) [76]. By inactivating endogenous PGE_2_, this enzyme provides a natural way of reducing the intracellular levels and regulating extracellular secretion of this lipid metabolite. Remarkably, both pharmacological inhibition of COX-2/mPGEs1 pathway and genetic restoration of 15-PGDH expression in myeloid cells could significantly reduce the tumor-mediated inhibitory effects on myeloid cells and improve their immune function [33, 75]. Together, these data indicate that aberrant PGE_2_ metabolism in tumor microenvironment markedly affects the immune function of tumor-infiltrating cells thus helping tumor cells to evade the immune response and promote tumor growth.

Epigenetic mechanisms seem to be responsible for the tumor-mediated deregulation of PGE_2_ metabolism in myeloid cells in cancer [77]. Thus, MDSCs isolated from patients with ovarian cancer displayed increased expression of DNA methyltransferase 3A (DNMT3A) levels that caused an extensive gene hypermethylation in PGE_2_-dependent manner. The hypermethylation signature in myeloid cells resulted in repression of specific genes associated with the immune response and promoted an immunosuppressive phenotype of myeloid cells. Down-regulation of DNMT3A levels markedly reduced MDSC-specific hypermethylation and attenuated their immunosuppressive function. We also previously reported [78] that treatment of tumor-infiltrating myeloid cells with DNA methylation inhibitor 5-aza-2′-deoxycytidine (5-AZA) promoted their differentiation toward mature APCs and markedly improved immune function. Collectively, these studies link PGE_2_-dependent DNA hypermethylation in cancer with tumor-associated inhibition of APC differentiation and concomitant accumulation of immunosuppressive myeloid cells.

There is a growing body of evidence demonstrating that PGE_2_ effectively inhibits DC differentiation while promoting macrophage and or MDSCs development, particularly in a combination with IL-6 [79–82]. Combination of PGE_2_ and IL-6 seems to be crucial for immune evasion in cancer. Thus, IL-6 alone could switch differentiation of DCs toward macrophages [82], and activates the transcription factor STAT3 which is required for up-regulation of PD-L1 expression [55]; while high levels of PGE_2_ inhibit DC differentiation, promotes accumulation of MDSCs and drives the cytokine profile toward the M_2_ phenotype in DCs/macrophages and Th_2_ type in T cells [68]. Altogether, these data indicate that enhanced and deregulated PGE_2_ metabolism in the bladder cancer promotes the formation of immunosuppressive tumor-supporting microenvironment.

Bladder cancer and tumor-infiltrating inflammatory cells in advanced tumors are positive for COX2 and exhibit increased expression of another PGE_2_-producing enzyme, mPGES1 [33]. High levels of PGE_2_ in tumor tissue have a strong impact on the function of infiltrating immune cells including the inhibition of APCs, effector T cell function and stimulation of MDSC generation directly through PGE_2_-specific EP2 and EP4 receptors. We have previously demonstrated that human bladder tumors secrete substantial amounts of PGE_2_ [39]. Culture of bone marrow-derived myeloid cells in PGE_2_-enriched bladder tumor-conditioned medium markedly inhibited the in vitro generation of mature APCs, while promoting the accumulation of monocytic MDSCs and macrophages.

## Emerging targets to remodel immunosuppressive microenvironment and improve the effects of bladder cancer immunotherapy

### CCR2/CCL2 axis

Chemokines and their receptors are involved in tumor progression by controlling cancer-related inflammation including the recruitment of immune cells to tumor tissue and lymphoid organs [83, 84]. Chemokine receptor 2 (CCR2) is a protein which represents one of the 19 chemokine receptors that are expressed predominantly by leukocytes. CCR2 expression was detected on monocytic myeloid cells including CD14 monocytes and its myeloid precursors, whereas its specific ligand CCL2 (MCP1) is produced by tumor and stromal cells. CCR2-expressing cells migrate to the source of CCL2 and are frequently recruited to tumor tissue, where they differentiate into tumor-promoting TAMs. In addition to the macrophage infiltration, the CCL2-mediated signaling axis has been implicated to the metastatic process in various cancers [85–89]. Targeting the CCR2–CCL2 axis reduced TAM accumulation at the metastatic site, thereby disrupting the immunosuppressive tumor microenvironment and improving the anti-tumor T-cell response. Notably, inhibition of CCL2 alone or in combination with anti-IL-6 therapy markedly reduced metastases and increased survival of the tumor-bearing animals [88].

In bladder cancer, CCL2/CCR2 interaction has been implicated in the stimulation of lymphoangiogenesis and development of lymphatic metastasis via macrophage tumor infiltration and VEGF-c production [90]. Mechanistically, the long noncoding RNA LNMAT1 epigenetically activates CCL2 expression via an activating promoter, which leads to increased histone methylation and enhanced VEGF-c transcription. In bladder cancer, myeloid cells isolated from peripheral blood secreted significant amounts of CCL2 constitutively [38]. Altogether, these findings provide a plausible mechanism for CCL2 in the recruitment of CCR2-expressing myeloid cells subsets to the tumor, thus promoting macrophage tumor infiltration and macrophage-mediated development of lymphatic metastasis. This suggests that the CCR2–CCL2 axis may represent a potential therapeutic target in bladder cancer.

### CCR8/CCL1, CCL18 axis

CCR8 belongs to the G protein-coupled receptor (GPCR) family. The natural ligands for CCR8 are CCL1 and CCL18. Expression of CCR8 is noted in several types of immune cells including T regs, monocytes, peritoneal macrophages, Langerhans cells and NK cells, but not in tumor cells [91–93]. Functionally, CCR8 has been implicated in cell migration, aggregation and cytokine production [94, 95] in response to cognate ligands. We previously demonstrated that monocytic and granulocytic myeloid cells obtained from peripheral blood and in tumor-infiltrating leukocytes of patients with bladder cancer display an increased expression of CCR8 [38]. Primary bladder cancers secrete substantial amounts of the natural CCR8 ligand CCL1. Remarkably, CCR8 expression detected in bladder cancer tissue and was limited to the tumor-infiltrating myeloid cells, including TAMs. We also noted that the tumor-infiltrating CD11b^+^CCR8^+^ cell subset responsible for the production of the greatest levels of the pro-inflammatory (IL-6, CCL3, CCL4) and pro-angiogenic (VEGF) factors among intra-tumoral myeloid cells. Furthermore, CD11b^+^CCR8^+^ cells were noted to induce FOXP3 expression in T lymphocytes [37].

Recent studies support a tolerogenic nature of CCR8-expressing cells and its ligand CCL1 [96, 97]. Thus, recombinant CCL1 in a dose-dependent manner is capable of inducing FOXP3 transcription factor in CD4 T cells. Furthermore, administration of anti-CCR8 antibody in mice with transplanted colorectal tumors significantly inhibited tumor growth [97]. The anti-tumor effects were accompanied with reduction of both, T regs and MDSCs. Given the increased levels of CCR8-expressing cells in patients with bladder cancer, CCR8 and/or CCR8-related ligands could represent an attractive target for therapy of bladder cancer.

### mPGES1

Increased COX2-dependent PGE_2_ production strongly contributes to the formation of an immunosuppressive and tumor-promoting microenvironment. Due to adverse effects of COX inhibitors on the cardiovascular system, stomach and kidney [98, 99], novel approached that target the increased and deregulated PGE_2_ production warranted. Microsomal PGE synthase 1 (mPGES1) is microsomal prostaglandin E synthase-1 (mPGES-1), the key terminal enzyme involved in the synthesis of PGE_2_. This is an integral membrane enzyme, which is highly expressed at sites of inflammation, and considered to be responsible for the excessive PGE_2_ synthesis and is suggested as a promising target for suppressing PGE_2_ biosynthesis [100, 101]. mPGES-1 gene knockdown in cancer cell lines resulted in decreased clonogenic capacity, slower growth and increased apoptosis of tumor cells, which could be rescued by exogenous PGE_2_ [102]. Since mPGES1 is critically involved in PGE_2_ production, it inevitably contributes to PGE_2_-mediated cancer-related inflammation and formation of immunosuppressive tumor microenvironment. Recent studies suggest that inhibition of mPGES1 activity resulted in reduced PD-L1 expression in myeloid cells infiltrating bladder tumor tissue [33]. Furthermore, expression of mPGES1 is necessary for induction of T regs and IL-17-producing T cells during primary immune response [103]. Altogether, these data indicate that targeting mPGES1 in bladder cancer could reduce the tumor-associated immunosuppression and improve the efficacy of cancer immunotherapy.

### Tumor-produced hyaluronan

Microenvironmental signals determine the differentiation and distinct function of macrophages. Hyaluronan or hyaluronic acid (HA) is a member of the glycosaminoglycan family of polysaccharides and a major component of the extracellular matrix (ECM). HA is synthesized at the cell surface as a large polysaccharide polymer with very high molecular weight (2 × 10^5^ to 10 × 10^7^ kDa) and extended length of 2–25 µm [104, 105]. HA is extruded through the plasma membrane onto the cell surface or into the ECM as it is being synthesized. HA is known to be a prominent component of the tumor microenvironment’s ECM, and bladder cancer is characterized by aberrant HA metabolism resulting in increased production in tumor tissue [106–108]. Membrane-bound or free extracellular HA favors tumor progression by inducing tumor cell motility, invasive properties, proliferation, production of growth factors and epithelial-mesenchymal transition [105]. HA is also involved in the regulation, proliferation and differentiation of hematopoietic cells in bone marrow [109].

In addition to the tumor cells, HA can also interact with CD44 receptors expressed by immune cells and other cells of hematopoietic origin. HA modulates expression levels of various cytokines and chemokines in macrophages [110–112]. Additionally, it has been noted that the effect of HA depends on its molecular weight. In general, high molecular weight HA has been shown to be anti-inflammatory and anti-angiogenic. In contrast, fragmented low molecular weight (LMW) HA stimulates expression of pro-inflammatory cytokines, chemokines and growth factors. Furthermore, LMW HA is a potent stimulant of cPLA2 promoting strong release of arachidonic acid, which is a substrate for the inflammation-associated enzymes COX2 and lipoxygenase [113]. In addition, increased levels of HA synthase in cancer tissue correlated with TAM count within tumor mass [114]. Collectively, deregulated HA metabolism in the tumor microenvironment may contribute to the cancer-related inflammation and immune evasion thus promoting tumor growth.

## Conclusion

Cancer immunotherapy is one of the most effective and promising modalities for cancer treatment. Novel insights into immunology and cancer biology have stimulated research efforts to bring novel immunotherapeutic agents into clinical practice. The last decade has been marked by significant progress in developing, clinical testing and validation of such agents, including immune checkpoint inhibitors, engineered immune cells and novel cancer vaccines. However, the clinical efficacy of cancer immunotherapy is limited due to tumor-induced immune suppression and immune tolerance. Therefore, simultaneous targeting of tumor-induced immune suppression and administration of immunotherapeutic agents has a great potential to boost the anti-tumor immune response and produce a more powerful therapeutic effect than immunotherapy alone. This strategy may be extremely valuable in patients with advanced localized or metastatic bladder cancer which is characterized by high numbers of circulating MDSCs, frequent up-regulation of PD-L1 expression and significant infiltration of tumor tissue with immunosuppressive cell subsets. Recent advances in understanding cellular and molecular mechanisms of immune evasion in bladder cancer provide us with a hope that the therapeutic effects of cancer immunotherapy could be enhanced by pharmacological agents that target specific immunosuppressive components of the tumor microenvironment.

## Abbreviations

APC: Antigen-presenting cell
BCG: Bacille Calmette–Guerin
CCR: C–C chemokine receptor
CCL2: Chemokine (C–C motif) ligand
CAR: Chimeric artificial receptor
COX: Cyclooxygenase
cPLA2: Cytosolic phospholipase 2
DC: Dendritic cell
DNMT: DNA methyltransferase
ECM: Extracellular cell matrix
FOXP3: Forkhead Box P3
HA: Hyaluronan
IL-6: Interleukin 6
IL-10: Interleukin 10
MMP: Matrix metalloproteinase
mPGES1: Microsomal prostaglandin E synthase 1
MDSC: Myeloid-derived suppressor cell
PD: Programmed death
PD-L1: Programmed death ligand 1
PGE_2_: Prostaglandin E2
STAT: Signal transducer and activator of transcription
tDC: Tolerogenic dendritic cell
TGF-β: Transforming growth factor-beta
TAM: Tumor-associated macrophage
VEGF: Vascular endothelial growth factor
